# Effect of wine pomace extract on dechlorination of chloroethenes in soil suspension

**DOI:** 10.1186/s40643-023-00643-6

**Published:** 2023-03-29

**Authors:** Takashi Ohashi, Kenji Sugimoto, Yoshikatsu Sasaki, Masashi Hisamoto

**Affiliations:** 1 NIPPO Corporation, 3-32-34 Higashi-Shinagawa, Shinagawa-ku, Tokyo 140-0002 Japan; 2 Department of Integrated Applied Life Science, University of Yamanashi, 4-4-37 Takeda, Kofu, Yamanashi 400-8510 Japan; 3 The Institute of Enology and Viticulture, University of Yamanashi, 1-13-1 Kitashin, Kofu, Yamanashi 400-0005 Japan

**Keywords:** Bioremediation, Dechlorination, Wine pomace, Chloroethene, Carboxylic acid

## Abstract

Chloroethenes are widely used as solvent in the metal industry and the dry cleaning industry, but their spillage into soil and groundwater due to improper handling has negatively impacted human health. Bioremediation using microorganisms is one of the technologies to clean up soil and groundwater contaminated with chloroethenes. In this study, we examined the bioremediation of chloroethene-contaminated soil using wine pomace extract (WPE). WPE is a liquid containing seven major carboxylic acids and other substances extracted from grape pomace produced in winemaking. WPE clearly promoted the anaerobic bioremediation of chloroethenes. In the tetrachloroethene (PCE) degradation test that used fractions derived from WPE, the water-eluted fraction containing l-lactic acid, l-tartaric acid, and others promoted the dechlorination of PCE, whereas the methanol-eluted fraction containing mainly syringic acid did not. In another PCE degradation test that used l-lactic acid, l-tartaric acid, and syringic acid test solutions, l-lactic acid and l-tartaric acid enhanced the dechlorination of PCE, but syringic acid did not. The results suggest that l-lactic acid and l-tartaric acid in WPE function as hydrogen donors in the anaerobic microbial degradation of chloroethene. This technology realizes environmental remediation through the effective use of food by-products.

**Graphical Abstract:**

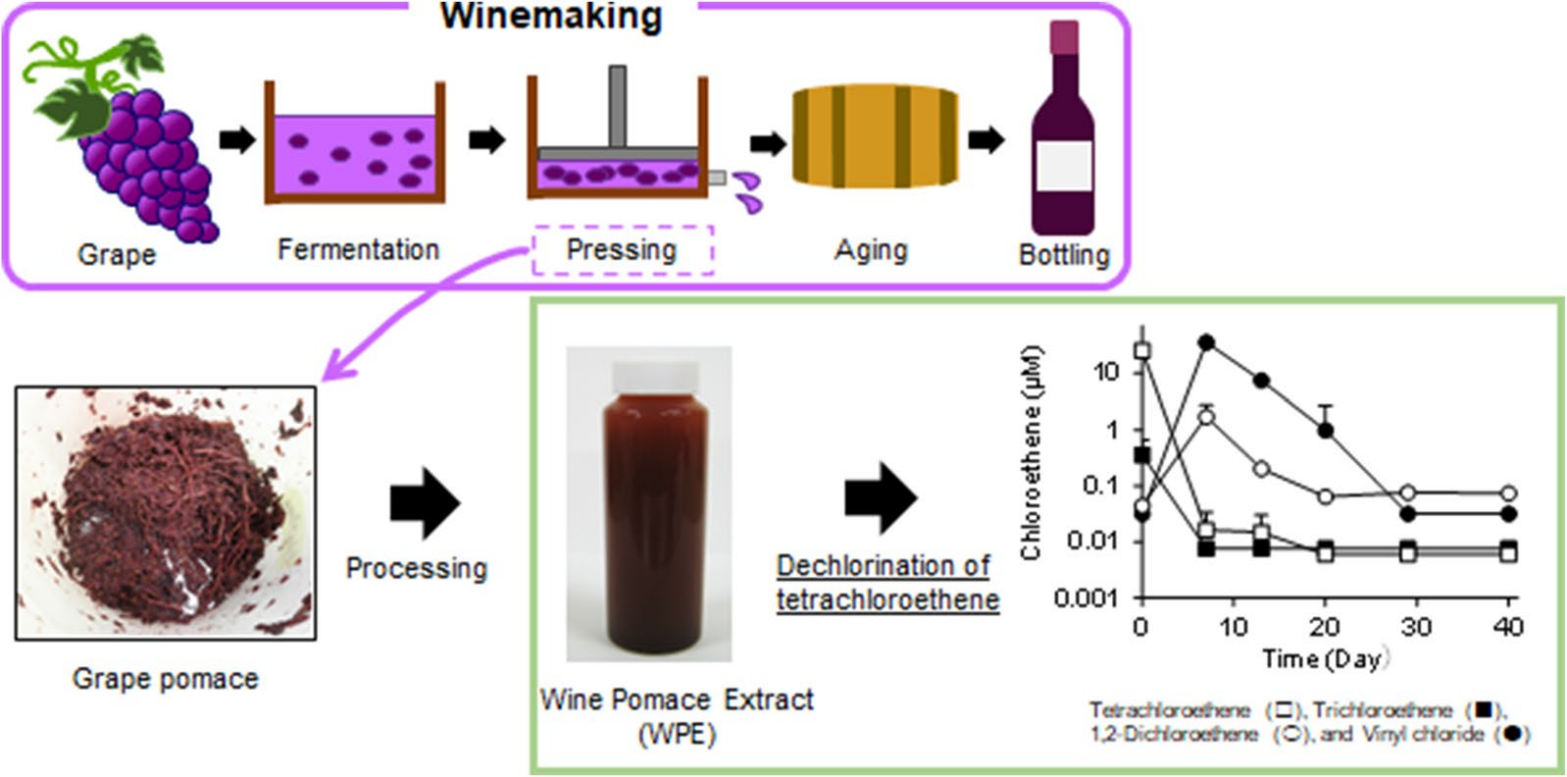

## Introduction

Chloroethenes such as tetrachloroethene (PCE) and trichloroethene (TCE) are widely used as solvent in the metal industry and the dry cleaning industry (McCarty 2010). According to the International Agency for Research on Cancer (IARC), TCE and vinyl chloride (VC) have the highest “strength of evidence” of carcinogenicity to humans and are, therefore, classified into Group 1 (IARC 2014). The contamination of soil and groundwater due to the careless handling and storage of chloroethenes has adversely affected human health (Moran et al. 2007). Groundwater and soil remediation technologies include physicochemical treatment methods, such as groundwater pumping techniques (Park 2016) and soil gas suction (USEPA 2012). However, these technologies require much cost and energy (Park 2016) and are meagerly effective for low concentrations of contaminants. In recent decades, bioremediation using microorganisms has been developed and put to practical use (Bradley 2003). Microbial reductive dechlorination, the main degradation pathway for chlorinated hydrocarbons in anaerobic subsurface environments, has been widely studied (Dolinová et al. 2017). Anaerobic microorganisms capable of degrading chloroethenes sequentially dechlorinate dichloroethene (DCE) and VC into ethene (ETH) using hydrogen generated from the degradation of hydrogen donors such as organic acids as the electron donor and chloroethenes such as PCE as the electron acceptor, thereby detoxifying them (Dolinová et al. 2017). Reductive dechlorination is a continuous electron transfer process in which hydrogen produced by hydrogen donors under anaerobic conditions is replaced by chlorine atoms of chloroethene. In this process, PCE is degraded via TCE and DCE isomers (mainly *cis*-1,2-DCE, *trans*-1,2-DCE, and 1,1-DCE) into VC and finally into harmless substances, such as ETH. Although it is known that many microorganisms can degrade TCE into DCE (Saiyari et al. 2018), *Dehalococcoides* spp. and a portion of *Propionibacterium* spp. were reported to degrade PCE and 1,2-DCE into ETH (Chang et al. 2011). Hydrogen donors include formic acid, acetic acid, glucose, methanol (Freedman and Gossett 1989; Pavlostathis and Zhuang 1993), and lactic acid (De Bruin et al. 1992) as single substrates and suspended vegetable oils (Newman and Pelle 2006), molasses, and whey (DiStefano et al. 2001; Macbeth et al. 2006). There are also commercially available polylactic acid-based products for the bioremediation of actual contaminated sites (Jin et al. 2005; Sandefur and Koenigsberg 1999). Wine pomace contains tartaric acid, lactic acid, malic acid, and phenolic acids as organic acids (Ribéreau-Gayon et al. 2021), which may function as hydrogen donors. Most of the hydrogen donors proposed so far are in the liquid form and thus tend to diffuse widely when injected into soil (Newman and Pelle 2006). Wine pomace cannot be injected into soil, because it is a solid that is insoluble in water. Nevertheless, we have confirmed that when wine pomace extract (WPE) was injected into soil, it spread at a rate equivalent to the velocity of groundwater (Ohashi et al. 2023). We have developed WPE containing organic acids and other substances extracted from wine pomace. WPE is prepared by solubilizing and liquefying wine pomace by making it alkaline with sodium hydroxide.

Grapes are a typical fruit crop produced worldwide. The berries are consumed fresh and as processed products. The most common processed product is wine. According to the International Organization of Vine and Wine, an intergovernmental organization, wine production in 2020 is estimated at 26 billion liters (OIV 2020). Wine pomace is grape pomace produced during wine production. Composed of grape seeds, skins, and stalks, wine pomace accounts for 20–30% of the total weight of grapes used in the winemaking process (Antonić et al. 2020). Although wine pomace is generally employed in the production of spirits (e.g., grappa), tartaric acid, and animal feed (Arvanitoyannis et al. 2006; Teixeira et al. 2014), it remains largely underutilized because of its unreliable quality, unstable supply, and aggregation difficulty. The use of wine pomace as biomass for energy and fuel and the effective utilization of unutilized bioactive substances such as polyphenols have been explored (Beres et al. 2017; Sirohi et al. 2020).

Research on the use of wine pomace for environmental remediation has been pursued in earnest (Kalli et al. 2018). There have been attempts to use wine pomace to treat toxic substances by converting it into porous carbon to adsorb heavy metals in wastewater (Nayak et al. 2016). Yang et al. have reported that pristine (not impacted by anthropogenic chlorinated solvents) habitats, such as grape pomace compost harbor organohalide-respiring bacteria (Yang et al. 2017). They have also suggested that *Dehalogenimonas* is likely a greater contributor to the reductive dechlorination of chlorinated solvents in contaminated aquifers than currently recognized. These studies are significant for the realization of a sustainable society. The objectives of this study are as follows: (a) to confirm that WPE contributes to the microbial degradation of chloroethenes; (b) to identify the types of carboxylic acids present in WPE; and (c) to verify whether the identified substances function in the microbial degradation of chloroethenes.

## Materials and methods

### Chemicals and stock solution

PCE (> 99% purity) and mixture standard solution (mixed standard solutions of 14 chloroethenes, including PCE, TCE, 1,2-DCE, and VC, in methanol solution (each 1 mg/mL methanol solution for soil pollutant analysis)) were purchased from FUJIFILM Wako Pure Chemical Co. (Osaka, Japan). The mixture standard solution was used as the standard for gas chromatography. All of the other chemicals used were reagent grade or higher unless otherwise specified. PCE saturated-water stock solution containing approximately 0.9 μmol of PCE per mL was added to cultured groundwater.

### Production of WPE

**Fig. 1:**
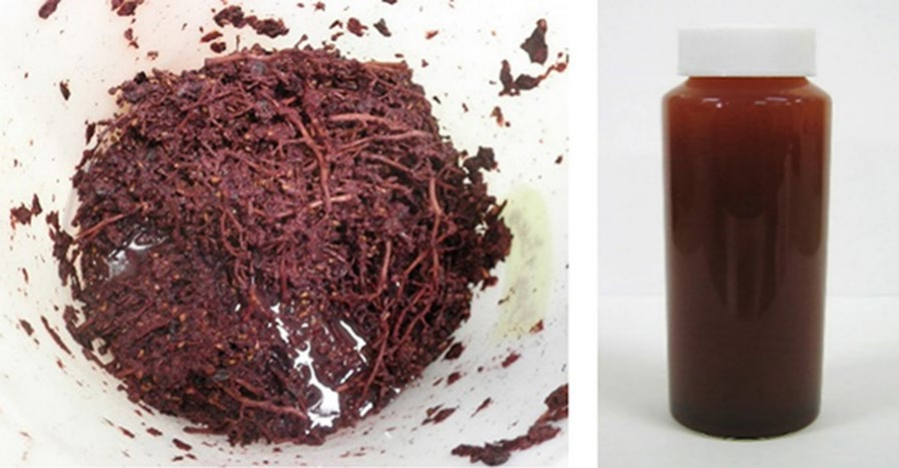
Wine pomace from the red wine production process (left) and its wine pomace extract (right)

Wine pomace was generated during red wine production using Muscat Bailey A grape. Muscat Bailey A is a hybrid grape variety [*Vitis labruscana* (Bailey) × *Vitis vinifera* (Muscat Hamburg)], and its red wine is one of the most popular in Japan. In this study, we used wine pomace collected from wineries in Yamanashi Prefecture in 2019. WPE was prepared by mixing 108 kg of wine pomace with 229 kg of tap water and 54 kg of 25% sodium hydroxide (food additive grade) and macerating for 4 weeks. After 4 weeks, the mixture was placed in a filter bag and pressed to separate undissolved solids. Then, 10% hydrochloric acid (food additive grade) was added to the liquid that passed through the filter bag to adjust the pH to approximately 2. The pH-adjusted liquid was allowed to stand for 3 days and after that, the supernatant was separated and collected. The weight of the supernatant was approximately 80% of the weight of the pH-adjusted solution. This supernatant was used as WPE in the following tests (Fig. 1).

### Chemical properties of WPE

The chemical properties of WPE were measured according to the Japanese Industrial Standard (JIS K0102, testing methods for industrial wastewater): pH by the glass electrode method (JIS K0102 12.1); total organic carbon (TOC) by the combustion oxidation-infrared TOC method (JIS K0102 22.1); ammonia nitrogen, nitrite nitrogen, and nitrate nitrogen by indophenol blue absorptiometry (JIS K0102 42.2, 43.2.1); phosphate phosphorus by molybdenum blue absorptiometry (JIS K0102 46.1); and suspended solids concentration (SS) by the suspended solids method (JIS K0102 14.1).

### Separation of WPE

WPE was separated on a reversed-phase column (Strata C18-E55 µm, 70 Å, 10 g/60 mL, Phenomenex, Inc., USA). The column was equilibrated with distilled water (three column volumes). Lyophilized WPE (28 mg) was separately loaded into the column and distilled water (three column volumes) was added to elute the organic acids. The eluate was used as the water-eluted fraction. Then, methanol (three column volumes) was added to obtain the methanol-eluted fraction. The two fractions were evaporated at reduced pressure and temperature (< 35 °C) and the extracts were lyophilized separately to yield 22 mg and 10 mg, respectively.

### Quantitative analysis of carboxylic acids in WPE using LC/MS/MS

Waters Acquity H-class UPLC systems coupled to Waters TQ-XS triple quadrupole mass analyzers (Waters Corporation, Wilmslow, UK) were employed. Chromatographic separation of the analytes was performed on an Acquity UPLC HSS T3 column (1.8 μm, 2.1 × 100 mm; Waters Corporation, Milford, MA, USA). The eluent used for the separation consisted of 0.1% (v/v) formic acid diluted with ultrapure water (A) and 0.1% (v/v) formic acid in acetonitrile (B). Flow rate was 0.3 mL/min and column temperature was maintained at 40 °C. The autosampler compartment was cooled to 15 °C and 5 μL was injected. The total run time was 10 min. For the first 3.5 min, the mobile phase was 99% solution A and 1% solution B; from 3.6 to 5.0 min, it was 100% solution B; and from 5.1 to 10 min, isocratic elution was maintained with 99% solution A and 1% solution B. The weak and strong washes were water/acetonitrile 70/30 (v/v), respectively. The samples were filtered through a 0.45-μm membrane filter.

A Xevo TQ-XS mass spectrometer operated in the negative ESI mode was used for the detection of all analytes. The mass spectrometer was operated in the selective reaction monitoring (SRM) mode for the quantification of all analytes. The *m/z* values were 149.0 → 86.9 for tartaric acid, 132.9 → 115.1 for malic acid, 89.0 → 43.0 for lactic acid, 190.9 → 111.1 for succinic acid, 162.9 → 118.8 for *p*-coumaric acid, 169.9 → 125.0 for gallic acid, and 196.9 → 122.9 for syringic acid. The final ion source settings were as follows: capillary voltage = 1.0 kV; cone voltage = 30 V; desolvation gas flow = 1000 L h^−1^; cone gas flow = 150 L h^−1^; nebulizer gas = 7.0 bar; desolvation temperature = 500 °C; and source temperature = 150 °C. WPE samples were analyzed in triplicate. The standard curves were drawn by measuring carboxylic acid standard solutions and were used to calculate the concentration of carboxylic acids in WPE. The MassLynx™ software, version 4.1 (Waters) was used for data acquisition and analysis.

### Methods for obtaining microbial community used in PCE degradation test

Soil-mixed groundwater collected from a TCE-contaminated site in Osaka, Japan was used as the microbial community capable of degrading chloroethenes. Soil-mixed groundwater was collected on September 24, 2019. At the time of sampling, pH was 6.7, electrical conductivity was 0.62 mS/cm, and the number of *Dehalococcoides* spp. was 1.1 × 10^4^ copies/mL. One liter of soil-mixed groundwater was added into a glass container and to this, WPE (6 mL) and PCE saturated-water stock solution (2 mL) were added. The mixture was incubated at 30 °C. Every few weeks, the mixture was transferred to another 100-mL container and 100 mL of soil-mixed groundwater collected from the same site was added repeatedly. In addition, every few months, WPE (6 mL) and PCE saturated-water stock solution (2 mL) were added. This cultured groundwater was used as the microbial community. Microorganisms with dechlorinating ability in the cultured groundwater were measured by real-time PCR targeting the 16S ribosomal RNA gene of *Dehalococcoides* spp. (He et al. 2003; Kurata et al. 2001).

### PCE degradation tests

Some of the PCE degradation tests were carried out as reported by He et al. (He et al. 2003). The PCE degradation tests were performed in a glass bottle (123 mL). To a glass bottle was added 700 µL of WPE dissolved to make a concentration of 28 mg/L, the water-eluted fraction or the methanol-eluted fraction derived from WPE, or 110 µL of each aqueous test solution (100 mM l-lactic acid solution, 100 mM l-tartaric acid solution, or 100 mM syringic acid solution). Then, 5.5 mL of soil suspension was added and the total volume was made to 107.4 mL by adding cultured groundwater. The soil suspension was prepared by adding 100 mL of sterile water to 30 g of soil collected from a chloroethene-contaminated site. The control was composed of the soil suspension and cultured groundwater only. The suspensions were sealed tightly with Teflon-lined butyl rubber stoppers and aluminum seals.

**Fig. 2:**
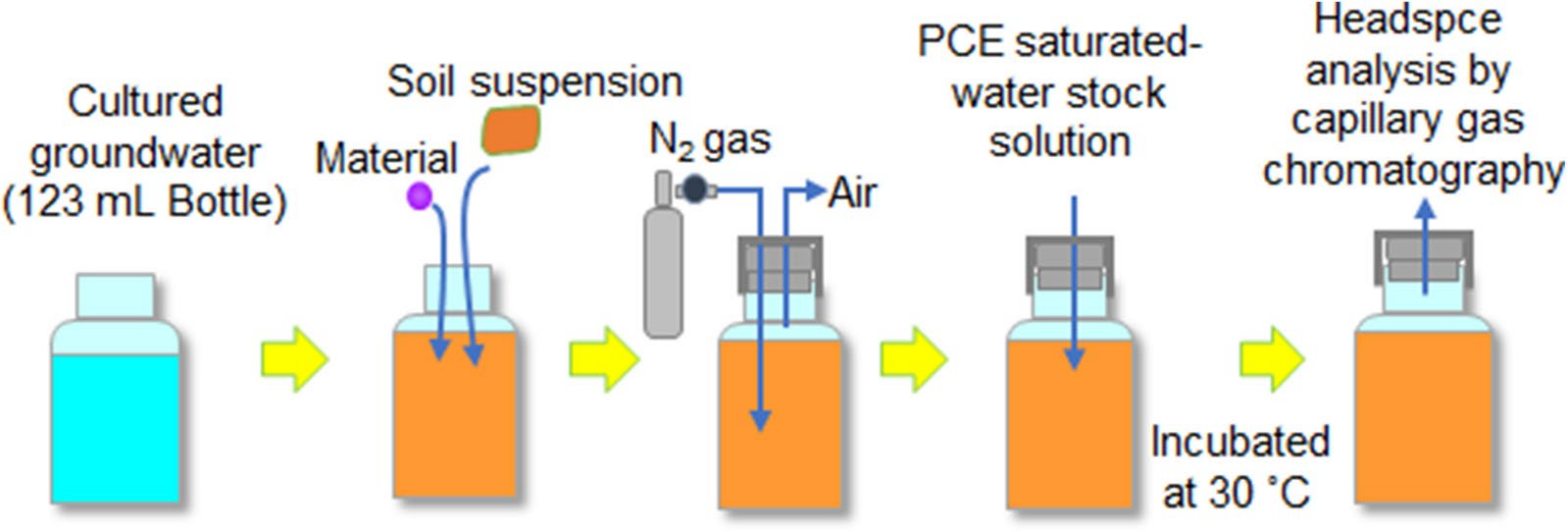
Schematic diagram of the PCE degradation test

The inside of the glass bottle was purged with nitrogen gas to create an anaerobic environment, and 2.6 mL of PCE saturated-water stock solution was injected into the glass bottle with a microsyringe. PCE saturated-water stock solution containing approximately 0.9 μmol of PCE per mL was added to cultured groundwater. In the PCE degradation test using the two fractions of WPE, the amounts added were 28 mg, 12 mg, and 10 mg for WPE, the water-eluted fraction, and the methanol-eluted fraction, respectively, according to the yields of the column fractionation. In the PCE degradation test with the three carboxylic acids, the final concentrations were 0.1 mM each for -lactic acid, l-tartaric acid, and syringic acid, and 22.7 μM for PCE. The glass bottle was allowed to stand at 30 °C and the concentrations of chloroethenes were measured at any given time (Fig. 2).

### Gas chromatography (GC)

The determination of chloroethenes (VC, 1,2-dichloroethene (1,2-DCE), TCE, and PCE) in culture medium in the glass bottle was carried out by modifying the method of Freedman and Gossett (Freedman and Gossett 1989). In this study, a headspace gas injection method using a gas chromatograph (GC-310C, Techno International, Japan) with a dry electrolytic conductivity detector (DELCD) was used. The sample was injected into a capillary column (UA-624, 30 m long, 0.53 mm i.d., 3.0 µm film thickness, Frontier Laboratories Inc., Japan) packed with 6% cyanopropylphenyl polysiloxane. Helium was used as the carrier gas; output from the DELCD was analyzed with PeakSimple chromatography software (SRI Instruments Inc.). The column oven was heated to 40 °C and held for 1 min at that temperature. Then, the temperature was increased at the rate of 8 °C per min to 72 °C, 5 °C per min to 100 °C, and 10 °C per min to 120 °C. To directly relate the GC peak areas obtained from 0.05 mL headspace gas injection to the masses of the compounds in the glass bottle, the GC calibration factor was determined. 1,2-DCE was the sum of *cis*-1,2-DCE and *trans*-1,2-DCE.

### Statistical analysis

Analysis of variance (ANOVA) was conducted using JMP™ (Version 17, SAS Institute, Cary, NC, USA) software. Significant (*p* < 0.05) differences between means of three replicates were identified using the Tukey (HSD) multiple comparison test.

## Results

### Chemical analysis of WPE

Table 1 shows the chemical properties of WPE. The pH value was adjusted to 2–3 during the production process. TOC, an indicator of organic matter content, averaged 16,000 mg/L; ammonia nitrogen averaged 150 mg/L; nitrite nitrogen and nitrate nitrogen averaged 37 mg/L; phosphate phosphorus averaged 56 mg/L; and SS averaged 280 mg/L. The pH of WPE is acidic, but because the amount of WPE added is small, the pH of the test solution during the PCE degradation test is 7–8. This pH does not inhibit the dechlorination ability of *Dehalococcoides* spp. The low pH of WPE is suitable for improving the shelf life of the product. TOC is an index to estimate the amount of organic matter contained in WPE. It is useful for observing the residual amount and spread of WPE at actual contamination sites. Nitrite nitrogen, nitrate nitrogen, and phosphate phosphorus, although necessary for microbial growth, were found to be present in small amounts in WPE. SS indicates the amount of soluble solids in WPE. Because high SS levels can block injection wells and hinder injection, we prepared our WPE, so that its concentration was lower than 500 mg/L for diffusion into soil.

**Table 1:** Chemical properties of wine pomace extract

Chemical property	Wine pomace extract^a^	
	Minimum	Maximum
pH	2.3	2.9
Total organic carbon (mg/L)	13,000	18,000
Ammonia nitrogen (mg/L)	60	240
Nitrite nitrogen and nitrate nitrogen (mg/L)	8	110
Phosphate phosphorus (mg/L)	20	93
Suspended solids (mg/L)	200	360

^a^The maximum and minimum measured values are shown for each batch of WPE produced eight times.

### Quantitative analysis of carboxylic acids in WPE

Wine pomace produced during winemaking contains carboxylic acids as potential hydrogen donors, particularly tartaric acid (Nurgel and Canbas 1998). Therefore, we analyzed the carboxylic acids contained in the WPE prepared in this study.

Table 2 shows the results of the quantitative analysis of carboxylic acids in WPE using LC/MS/MS. Seven carboxylic acids were present in the WPE in decreasing order of content: l-lactic acid, l-tartaric acid, succinic acid, *p*-coumaric acid, syringic acid, l-malic acid, and gallic acid.

**Table 2:** Concentrations of carboxylic acids in wine pomace extract by LC/MS/MS

Carboxylic acid	Concentration (µM)
l-Lactic acid	25,000 ± 1400
l-Tartaric acid	17,000 ± 600
Succinic acid	2800 ± 140
*p*-Coumaric acid	510 ± 2.5
Syringic acid	330 ± 0.68
l-Malic acid	170 ± 10
Gallic acid	120 ± 2.1

Data are means ± SDs of three individual estimates

### PCE degradation test using WPE, fractions derived from WPE, and carboxylic acid test solutions

**Fig. 3:**
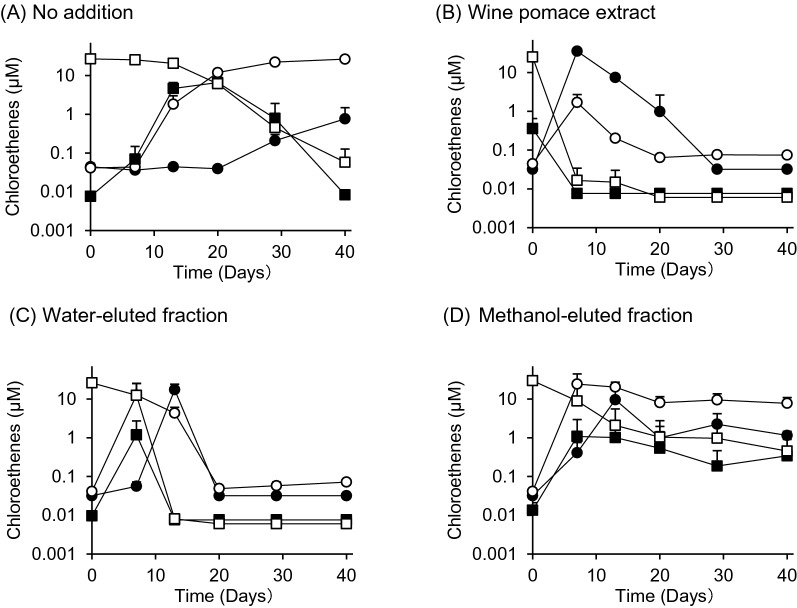
Dechlorination of tetrachloroethene into ethene in cultured groundwater by wine pomace extract or two fractions of wine pomace extract. Symbols in the graphs represent the concentrations of tetrachloroethene (open squares), trichloroethene (closed squares), 1,2-dichloroethene (open circles), and vinyl chloride (closed circles) over the 40-day test period. Values are means ± SD (*n* = 3)

Initially, the PCE degradation test was conducted on WPE and the fractions obtained from the column fractionation of the WPE. Even though no WPE or its fractions were added (No addition), the degradation of PCE and TCE proceeded and their degradation products, 1,2-DCE and VC, were formed (Fig. 3).

In the PCE degradation test using fractions derived from WPE, VC was generated by the degradation of PCE in the water-eluted fraction, but its concentration was reduced thereafter. On the other hand, in the methanol-eluted fraction, VC was generated by the decomposition of PCE, but its concentration was not reduced thereafter. The observed reduction in VC concentration with the addition of WPE or the water-eluted fraction may be due to the decomposition of VC into ETH as previously reported (He et al. 2003; Maymó-Gatell et al. 1997). LC–MS analysis indicated that the water-eluted fraction contained substantial amounts of l-lactic acid and l-tartaric acid. On the other hand, the methanol-eluted fraction contained syringic acid, gallic acid, and *p*-coumaric acid.

**Fig. 4:**
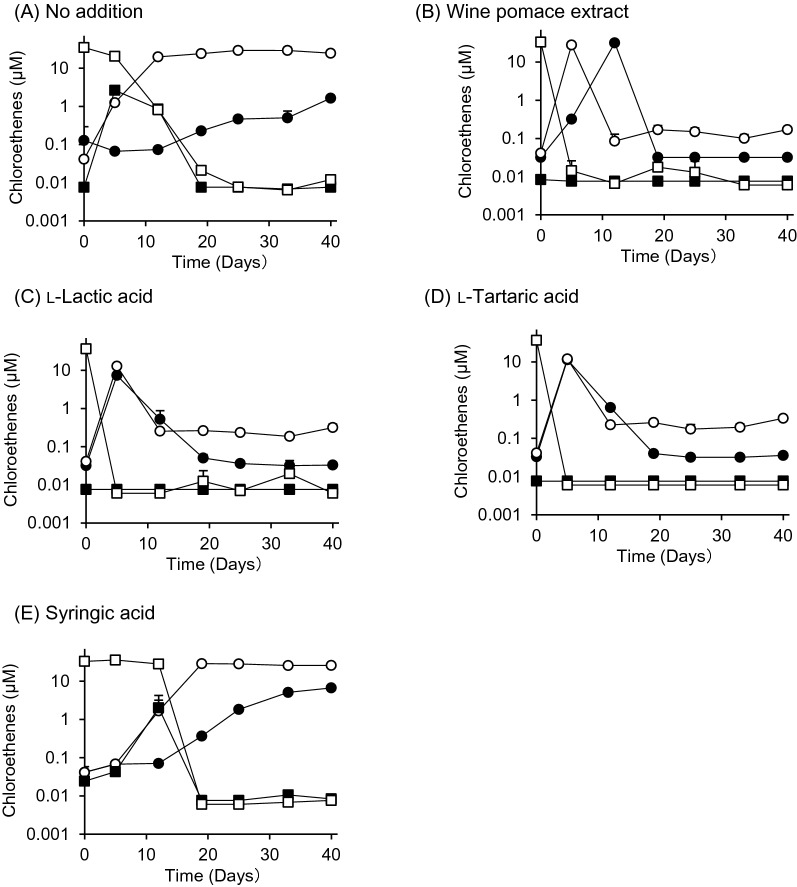
Dechlorination of tetrachloroethene into ethene in cultured groundwater by wine pomace extract or three carboxylic acids, i.e., l-lactic acid, l-tartaric acid, and syringic acid. Symbols in the graphs represent the concentrations of tetrachloroethene (open squares), trichloroethene (closed squares), 1,2-dichloroethene (open circles), and vinyl chloride (closed circles) over the 40-day test period. Values are means ± SD (n = 3)

Another PCE degradation test was conducted using WPE, l-lactic acid, l-tartaric acid, and syringic acid. Because the final concentration of l-lactic acid in the PCE degradation test using WPE was 0.1 mM, the final concentrations of l-tartaric acid and syringic acid were also set to 0.1 mM. Figure 4 shows the results. *Dehalococcoides* spp. were present in cultured groundwater at 1.1 × 10^4^ copies/mL at the beginning of the test.

In the no addition case, although the sequential degradation of PCE into TCE and 1,2-DCE proceeded, the increase in 1,2-DCE concentration plateaued after 10 days, and there was a slight increase in the concentration of VC, the degradation product of 1,2-DCE, from day 10 onward (Fig. 4A). On the other hand, when WPE was added, PCE concentration decreased abruptly, whereas the concentration of 1,2-DCE increased during the same period. The increase in the concentration of VC occurred slightly later than that of 1,2-DCE. 1,2-DCE concentration decreased to its lowest level on day 10, whereas VC concentration peaked on day 10 and decreased thereafter (Fig. 4B). When l-lactic acid and l-tartaric acid test solutions were added, PCE reached the lower limit of quantification (0.006 µM) around day 5, whereas 1,2-DCE and VC peaked on day 5 and quickly decreased thereafter (Fig. 4C, D). In the case of syringic acid addition, the degradation of PCE was delayed compared with that in the control case. In the control case, PCE was reduced to 1/42 of its initial concentration in approximately 12 days. On the other hand, in the case of syringic acid addition, PCE was reduced to 1/1.32 of its initial concentration on day 12, whereas TCE and 1,2-DCE were formed (Fig. 4E).

**Fig. 5:**
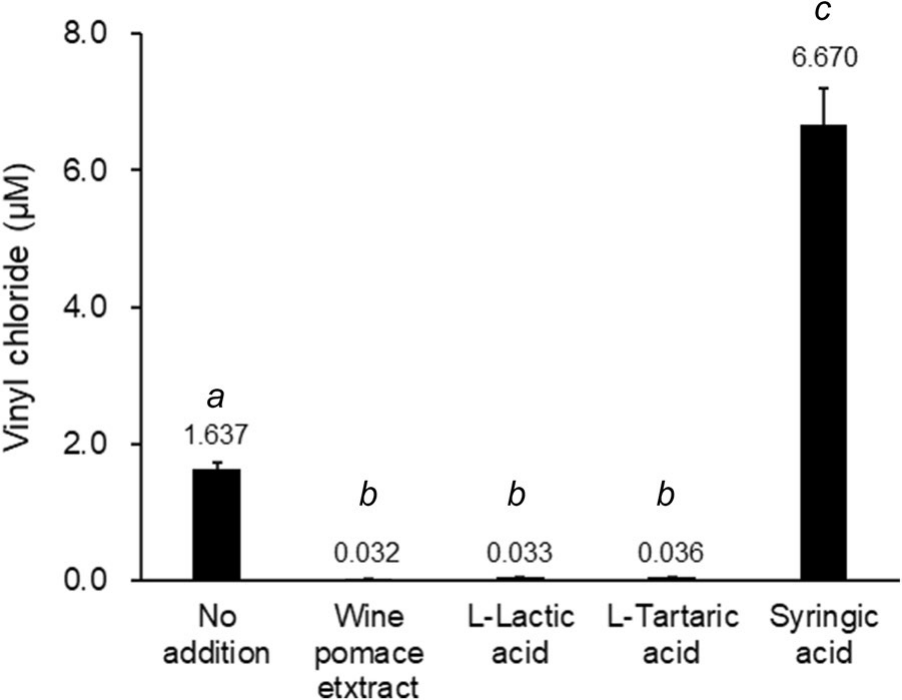
Concentrations of vinyl chloride on day 40 after adding WPE, l-lactic acid, l-tartaric acid, or syringic acid in the PCE degradation test. The result for the control (no addition) sample is also shown for comparison. Data are means of triplicate determination. ANOVA was performed to compare data. Values with different letters within each row are significantly different (Tukey’s test, *p* < 0.05)

Figure 5 shows the concentrations of VC on day 40 after adding WPE, l-lactic acid, l-tartaric acid, or syringic acid in the PCE degradation test. The result for the control (no addition) sample is also shown for comparison. The concentrations of VC in WPE, l-lactic acid, and l-tartaric acid added samples were significantly lower than that in the control sample. There were no significant differences in the concentrations of VC among WPE, l-lactic acid and l-tartaric acid added samples. WPE promoted the degradation of VC in the same manner as l-tartaric acid and l-lactic acid.

## Discussion

### Chemical properties of WPE

The composition of grapes may vary depending on extrinsic factors, such as edaphoclimatic conditions and viticultural practices, as well as intrinsic factors, such as variety, maturity, and sanitary conditions. Similarly, both the type of process and the conditions under which winemaking is carried out notably influence the composition of wine pomace. In this study, WPE was produced from wine pomace produced from the same vinification method each year at a particular winery to ensure consistency in the composition of wine pomace.

Various methods have been proposed to efficiently extract carboxylic acids from wine pomace, including chemical extraction using organic solvents, acid/alkali extraction, and physical extraction by ultrasound and crushing (Antonić et al. 2020; El Achkar et al. 2018; Filippi et al. 2021), but there is no consensus on which extraction method is best. In this study, carboxylic acids were extracted from wine pomace by alkaline maceration.

Alkaline maceration removes lignin from grape skins and stalks (Filippi et al. 2021), thereby softening the skins and stalks, and the skins and stalks are easily separated from WPE during the subsequent pressing process. l-Tartaric acid, succinic acid, and malic acid are found between the skin and pulp.l-Lactic acid is derived from l-malic acid via malolactic fermentation in the winemaking process. Because l-malic acid concentration is lower than l-lactic acid concentration, it is assumed that the wine pomace used in this study was recovered after alcoholic and malolactic fermentations were conducted simultaneously and malolactic fermentation had sufficiently advanced. When wine pomace is recovered after alcoholic fermentation followed by malolactic fermentation, l-malic acid concentration in the recovered wine pomace is higher than l-lactic acid concentration.*p*-Coumaric acid, syringic acid, and gallic acid are normally present in grapes in the form of carboxylic acid esters. These substances may have been extracted by the alkaline maceration of wine pomace. Their contents in grapes tend to be lower than that of l-lactic acid and l-tartaric acid, and the concentrations of carboxylic acids in WPE are consistent with this tendency (Table 2).

The fact that l-lactic acid concentration in WPE was 25 mM and TOC of 50 mM l-lactic acid solution prepared from reagent-grade l-lactic acid was approximately 1300 mg/L (data not shown) indicates that TOC of l-lactic acid in WPE was approximately 650 mg/L. Considering the presence of other carboxylic acids, TOC of WPE is high, ranging from 13,000 to 18,000 mg/L (Table 1). This TOC refers to the concentration of organic matter, and hydrogen donors are often organic matter. In fact, WPE contains carbohydrates (1.5 g/L by the phenol–sulfuric acid method) and amino acids (302 mg/L by the automatic amino acid analyzer), which may have functioned as hydrogen donors other than carboxylic acids. The effect of sugars and amino acids in WPE on the dechlorination of chloroethene should be investigated in the future.

### PCE degradation test using WPE and carboxylic acids

When only cultured groundwater was used (control), the sequential degradation of PCE stopped at 1,2-DCE. When WPE was added to the cultured groundwater, the degradation of 1,2-DCE into VC proceeded rapidly (Fig. 3). *Dehalococcoides* spp. and a portion of *Propionibacterium* spp. were reported to degrade PCE and 1,2-DCE into ETH (Chang et al. 2011), suggesting that WPE functioned as a hydrogen donor for these microbes and promoted the reductive dechlorination of PCE.

The reduction in VC concentration observed with the addition of WPE may be due to the degradation of VC into ETH, as previously reported (He et al. 2003). The seven carboxylic acids in WPE can be classified into two groups on the basis of their chemical structures: l-lactic acid, l-tartaric acid, succinic acid, and l-malic acid, which have aliphatic skeletons; and syringic acid, fumaric acid, and gallic acid, which have aromatic skeletons. In this study, not only l-lactic acid but also tartaric acid promoted the microbial dechlorination of PCE (Fig. 3), suggesting that succinic acid and l-malic acid, which are also aliphatic compounds in WPE, have a similar promotive effect to l-lactic acid.

Men et al. (2012) have reported that acetic acid and hydrogen produced by *Dehalococcoides* symbionts during the metabolism of l-lactic acid are continuously supplied to *Dehalococcoides* spp. at moderate concentrations to promote the dechlorination into chloroethenes. Similar to this report, l-lactic acid, l-tartaric acid, succinic acid, and l-malic acid in WPE may have been metabolized to produce hydrogen, which is important for the dechlorination process.

In contrast, syringic acid, a phenolic acid, more slowly degrades PCE than l-lactic acid or l-tartaric acid (Fig. 4). Syringic acid is formed by the alkaline hydrolysis of anthocyanins in wine pomace derived from red wine (Forester and Waterhouse 2008).

This may be because l-lactic acid and l-tartaric acid have aliphatic skeletons, whereas syringic acid has an aromatic skeleton that is not easily decomposed by anaerobic microorganisms, resulting in a low supply of hydrogen. Our finding that PCE was more slowly degraded in the syringic acid test solution than in control requires further verification.l-Lactic acid and WPE are comparable in terms of degradation rate and concentration reduction as hydrogen donors for the microbial degradation of PCE. Hydrogen Release Compound (HRC™), a commercially available hydrogen donor based on polylactic acid, provides lactic acid as the hydrogen donor (Jin et al. 2005; Sandefur and Koenigsberg 1999). WPE is as effective as HRC™ in acting as a hydrogen donor for the microbial degradation of chloroethenes.

The microflora degrading chloroethenes in the groundwater environment of actual contaminated sites differs from one contaminated site to another. This may result in degradation failure by bioremediation that uses only one hydrogen donor. Gibson and Sewell studied the effect of the addition of common fermentation products on the dehalogenation of PCE in a methanogenic slurry prepared from aquifer solids (Gibson and Sewell 1992). They reported that lactic acid, propionic acid, crotonate, butyrate, and ethanol stimulated dehalogenation, whereas acetate, methanol, and isopropanol did not (Gibson and Sewell 1992). Kengen et al. found that dechlorination was enabled by lactate, pyruvate, fructose, fumarate, and malate as electron donors, but not by hydrogen, formate, or acetate (Kengen et al. 1999). Santharam et al. conducted a pilot field study of the remediation of a PCE-contaminated site and reported that a mixture of soybean oil methyl esters, lactic acid, and yeast extract was effective for the remediation (Santharam et al. 2011). WPE contains not only carboxylic acids, such as organic and phenolic acids, but also sugars and amino acids. It has been reported that sugars and amino acids promote the bioremediation of chloroethenes (DiStefano et al. 2001; Zhuang et al. 2011). Therefore, it is suggested that WPE containing a variety of hydrogen donors is versatile, because any of its hydrogen donors would be effective for the anaerobic degradation of chloroethenes at actual contaminated sites, where the growth conditions of chloroethene-degrading bacteria vary.

## Conclusion

WPE is a liquid containing seven major carboxylic acids and other substances extracted from grape pomace produced in winemaking. WPE clearly promoted the anaerobic bioremediation of chloroethenes. Our results suggest that l-lactic acid and l-tartaric acid function as hydrogen donors in the anaerobic microbial degradation of chloroethene. This technology realizes environmental remediation through the effective use of food by-products.

## Data Availability

All data generated or analyzed during this study are included in this article.

## Abbreviations

WPE: Wine pomace extract
PCE: Tetrachloroethene
TCE: Trichloroethene
1,2-DCE: 1,2-Dichloroethene
VC: Vinyl chloride
